# A novel phenotype-genotype correlation with an Arg555Trp mutation of *TGFBI* gene in Thiel-Behnke corneal dystrophy in a Chinese pedigree

**DOI:** 10.1186/s12886-015-0121-0

**Published:** 2015-10-13

**Authors:** Yinhui Yu, Peijin Qiu, Yanan Zhu, Jinyu Li, Menghan Wu, Buyi Zhang, Ke Yao

**Affiliations:** Eye Center, Second Affiliated Hospital of Zhejiang University School of Medicine, No.88 Jiefang Rd, Hangzhou, 310009 China; Zhejiang Provincial Key Laboratory of Ophthalmology, Hangzhou, China; Department of Pathology, Second Affiliated Hospital of Zhejiang University School of Medicine, Hangzhou, China

**Keywords:** Genotype–phenotype, Thiel-Behnke corneal dystrophy, *TGFBI* gene, Mutation

## Abstract

**Background:**

To investigate the molecular defects in a four-generation Chinese pedigree affected with Thiel-Behnke corneal dystrophy (TBCD). And to further study the relationship between genetic mutation and clinical manifestations.

**Methods:**

Individuals of the pedigree were recruited for extensive ophthalmic examinations. Histological studies of two corneal buttons obtained from lamellar keratoplasty were conducted. Peripheral blood was collected in EDTA for genomic DNA isolation from leukocytes of all affected and unaffected members. All 17 exons of the *TGFBI* gene were screened for mutations by polymerase chain reaction and direct DNA sequencing.

**Results:**

Clinical examinations revealed a typical pattern of honeycomb-like TBCD. Histopathology study demonstrated eosinophilic deposits that were congo-red-positive and did not stain with periodic acid Schiff or Masson’s trichrome. Genetic analysis disclosed a heterozygous p. Arg555Trp mutation resulted from a missense c. 1663C > T nucleotide change in exon 12 of *TGFBI* gene in all affected members. Morever, a second rare variant in exon 6 of the *TGFBI* gene (p. Arg257Trp) also cosegregated within this family and has been confirmed to be a single nucleotide polymorphism (SNP) not previously reported.

**Conclusions:**

The p. Arg555Trp mutation of the *TGFBI* gene was associated with TBCD, which revealed a novel phenotype-genotype correlation within the mutational spectrum of phenotypically diverse corneal dystrophies.

## Background

Corneal dystrophies (CDs) belongs to a group of inherited disorders characterized by progressive accumulation of insoluble deposits at various depths of cornea. These variable opacities often result in loss of corneal transparency and serious visual impairment [1]. Since the initial discovery by Munier et al. [2, 3] of mutations in the transforming growth factor-beta induced (*TGFBI*; formerly designated as *BIGH3*) gene located on chromosome 5q31 that cause autosomal dominant CDs, several phenotype-specific mutations have been established, which include p. Arg555Trp in granular corneal dystrophy type I (GCD1), p. Arg124His in granular corneal dystrophy type II (GCD2), p. Arg124Cys in lattice corneal dystrophy type 1 (LCD1), p. Arg555Gln in Thiel-Behnke corneal dystrophy (TBCD) and p. Arg124Leu in Reis-Bücklers corneal dystrophy (RBCD) [4]. Among these, argnine residues at positions 124 and 555 account for more than 50 % of mutations associated with *TGFBI*-linked CDs.

Thiel-Behnke corneal dystrophy (TBCD, OMIM 602082) was first described in 1967 which is characterized by recurrent painful corneal erosions, moderately reduced vision, autosomal dominant inheritance, and honeycomb-shaped opacities at the level of Bowman’s layer [5]. To our knowledge, TBCD was most frequently correlated with the p. Arg555Gln mutation [6, 7], and the association of p. Arg555Trp mutation with TBCD has never been reported previously. Herein, we present the clinical, histological and genetic results of a Chinese pedigree affected with TBCD and identify a novel genotype-phenotype association within *TGFBI*-linked CDs.

## Methods

### Patients recruitment and evaluation

**Fig. 1:**
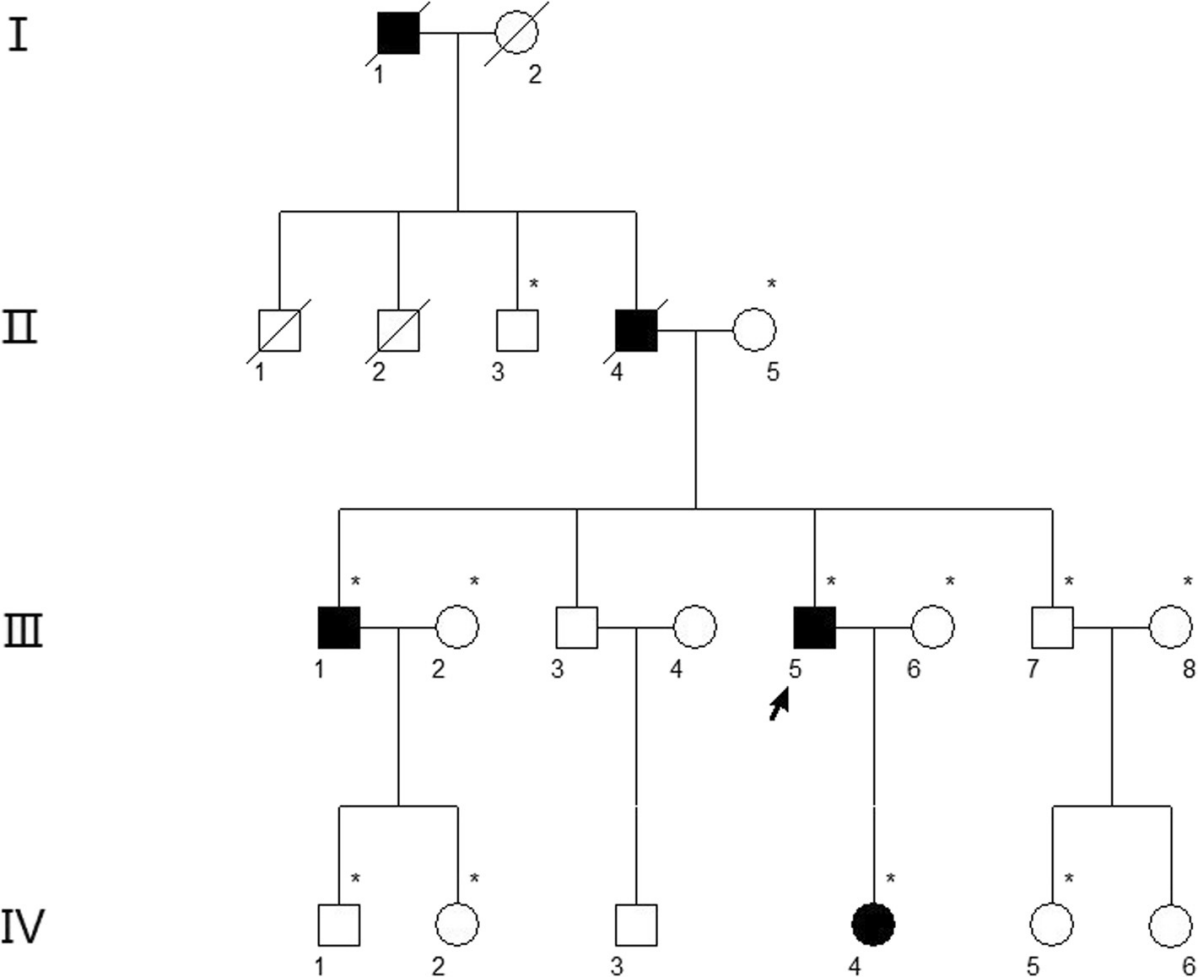
Pedigree of the four-generation Chinese family with autosomal dominant corneal dystrophy. Squares and circles indicate males and females, respectively. Black symbols indicate affected members and open symbols indicate unaffected individuals. The diagonal line indicates a deceased family member and the black arrow indicates the proband. Asterisks indicate family members who attend this study

Individuals from a four-generation pedigree of Chinese ethnicity with corneal dystrophy (Fig. 1) were recruited from the Eye Center of the Second Affiliated Hospital, Medical College of Zhejiang University, Hangzhou, China. The study adhered to the tenets of the Declaration of Helsinki, and has been aproved by the ethics committee of the 2^nd^ Affiliated Hospital of Zhejiang University.

We received informed written consent covering DNA extraction and analysis, as well as ocular and ophthalmological examinations from all participating individuals including 150 normal controls. The written consent of the 6-year old patient for participation was obtained by her farther. Full ocular examinations including Snellen visual acuity, slit-lamp examination, applanation tonometry, corneal sensation and anterior segment photography were conducted. In addition, the patients underwent profound ophthalmologic examinations including ultrasound biomicroscopy (UBM) and anterior-segment optical coherence tomography (AS-OCT) to assess depth of the lesions.

### Histological examination

Two corneal specimens of the proband were obtained from lamellar keratoplasty and underwent histopathological study. The buttons were processed by standard methods including sectioning of the tissue samples, fixation in 10 % neutral buffered formalin, then stained with hematoxylin and eosin (H&E), Congo red, Periodic acid-Schiff (PAS) and Masson’s trichrome, and analysis by light microscopy to detect the presence of deposits in the cornea.

### Molecular genetic analysis

Genomic DNA was extracted from the peripheral leukocytes of 12 members (three affected and nine unaffected) according to the manufacturer’s (Simgen, Hangzhou, China) standard methods. All 17 exons of *TGFBI* were amplified by a polymerase chain reaction (PCR) and analyzed with the molecular method described by Zhu et al. previously [8]. PCR products were separated by electrophoresis in a 1.5 % ethidium bromide-stained agarose gel. The bands with the amplified templates were examined, and then, the purified products were sequenced according to protocols accompanying the ABI Big dye Terminator Cycle sequencing kit V 3.1 (ABI Applied Biosystems; Foster City, CA). The nucleotide sequences were analyzed by using Lasergene V. 80 software (DNASTAR, Inc., Madison, WI) and compared manually with the published NCBI *TGFBI* cDNA sequence (GeneBank accession no. NM. 000358). DNA from 150 normal, unrelated, ethnically Chinese volunteers (75 males and 75 females) was analyzed as the control.

## Results

### Phenotyping

#### Case III-5 (the proband)

**Fig. 2:**
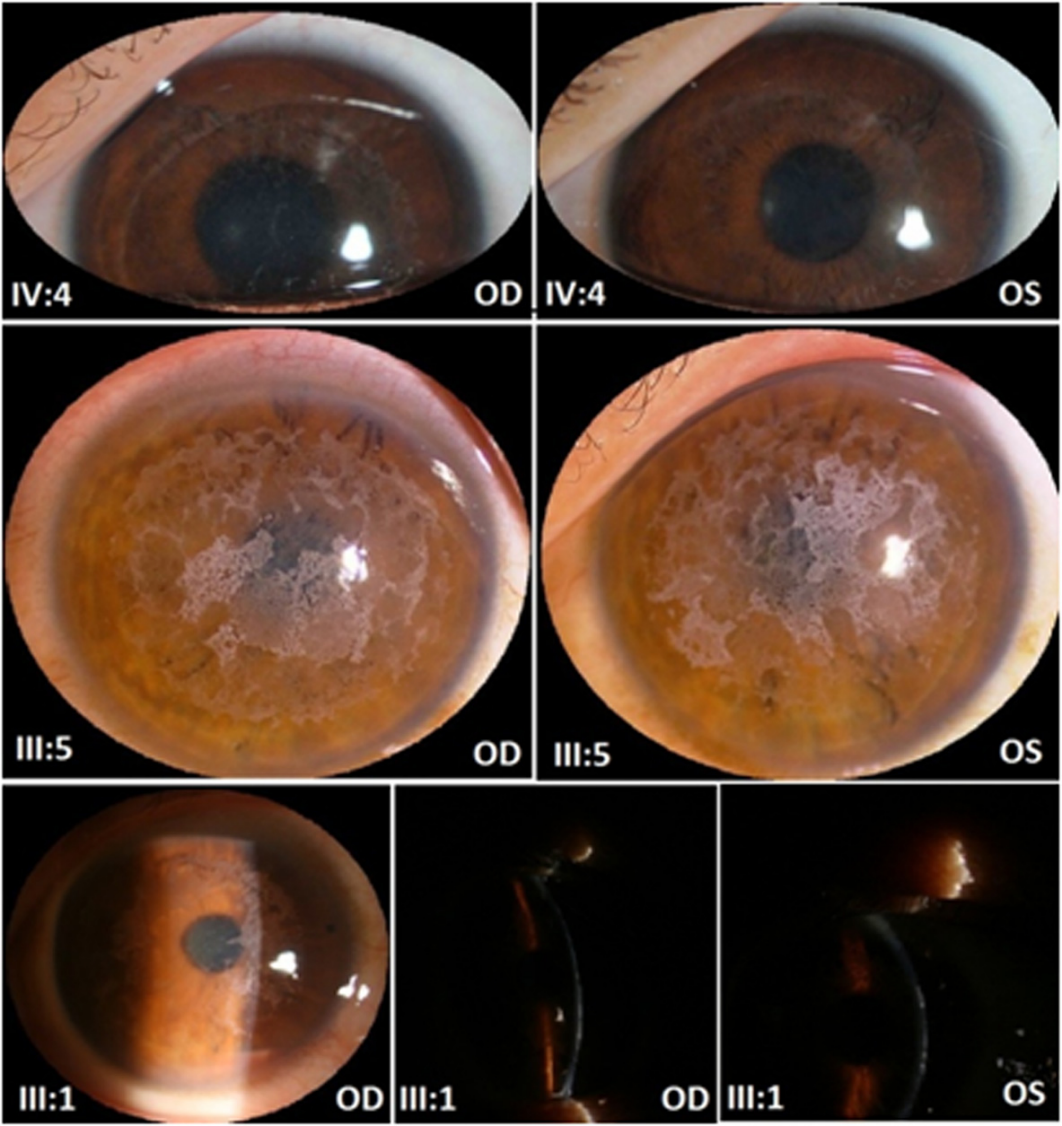
The affected family members’ corneal phenotype illustrating TBCD as shown by slit-lamp examination. Note the tiny superficial opacities of the proband’ daughter (IV:4), bilateral irregular corneal surfaces, multiple discrete, dense gray-white, honeycomb-shaped opacities in a geographic distribution of the proband (III:5), and extensive honeycomb-shaped configuration of grayish opacities beneath the epithelium and at the Bowman’s layer of the proband’s brother (III:1). Clear area appears around the corneoscleral limbus in all the patients. These images are published with the consent of the patients, and in the case of the 6-year old patient, with the consent of her parents

The 38-year-old male attended the outpatient clinic of our Eye Center in March 2012. He had complained of a history of recurrent, painful corneal erosions and a progressive decrease in visual acuity since he was 3 years old. Initial slit-lamp examination showed bilateral irregular corneal surfaces and discrete, dense, gray-white, honeycomb-shaped opacities in a geographic distribution involving all areas of cornea except the peripheral margin (Fig. 2). Ophthalmological exploration found a visual acuity of 0.05 in both eyes. Taken together, clinical findings support a diagnosis of TBCD. This led to bilateral lamellar keratoplasty, with an interval of five months between procedures. His Best Corrected Visual Acuity (BCVA) improved to 0.6 (OD) and 0.5 (OS) postoperatively. No episodes of rejection or recurrent dystrophic erosion have appeared thereafter with a follow-up interval of approximately 1 year.

#### Case IV-4 (the proband’ daughter)

The 6-year-old Chinese girl, was referred for intermittent attacks of ocular irritation and there was no family history of consanguinity. Her Uncorrected Visual Acuity (UCVA) was 1.0 in both eyes. Silt-lamp examination disclosed bilateral tiny superficial opacities that were prominent in whole cornea except the limbus (Fig. 2).

#### Case III-1 (the proband’s brother)

The 40-year-old man had complained of a very similar clinical course with his brother, and consistently showed an extensive bilateral honeycomb-shaped configuration of grayish opacities beneath the epithelium and at the Bowman’s layer (Fig. 2). Also observed were 3- to 4-mm peripheral clear zones. His BCVA was 0.12 (OD) and 0.15 (OS).

#### Case II-4 and Case I-1 (the proband’s father and grandfather)

The proband said these two affected patients had a history of intermittent ocular irritation, and both died blind.

### Histopathologic analysis

**Fig. 3:**
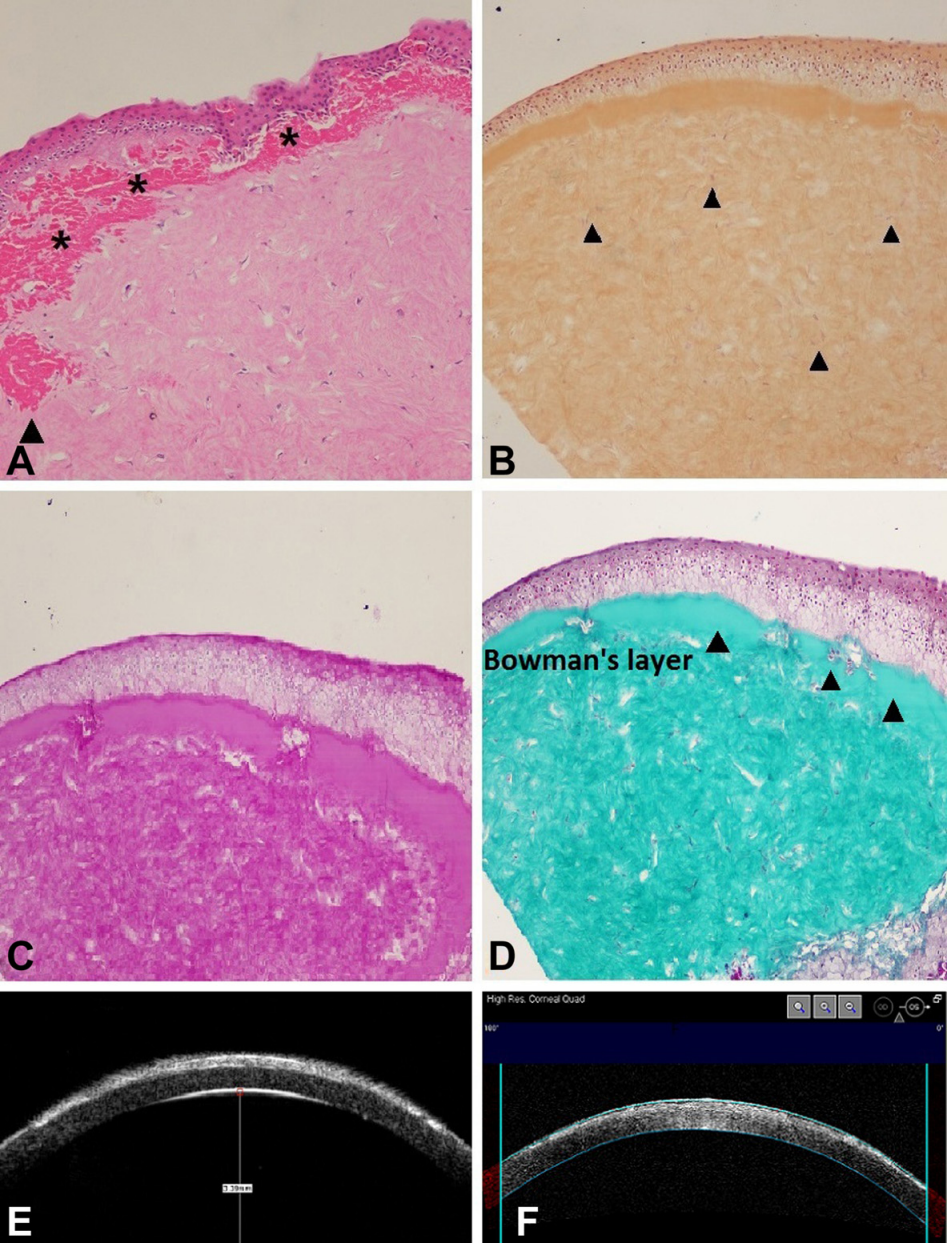
Histopathologic examination of the excised corneal button from the proband. **a** Hematoxylin and eosin staining of the tissue section revealed an irregular, layered structure of the corneal epithelium with variable thickness in which eosinophilic deposits (asterisks) were noted predominantly beneath the epithelium and involved the anterior stroma within a small range (see the arrow). Note the obliterated Bowman’s layer and distorted epithelium with varied thickness. **b** Orange-coloured congo-red-positive deposits of amyloid aggregates with variable size and irregular shape are found in subepithelia area, extending into the entire stroma (arrowhead). **c** Numerrous small single Periodic acid Schiff (PAS) stain-negative deposits were noted throughout the corneal stroma, which disturbed the normal architecture of the stroma. **d** The deposits did not stain red with Masson’s trichrome. Note the apparent subepithelial fibrosis. **e** UBM scan demonstrated markedly increased reflectivity, due to deposits within the lesions and irregular epithelial thickening induced by corneal opacities which project into anterior stroma. **f** OCT-scan also confirmed an extensive sawtooth-like pattern of hyperreflective material deposited along the Bowman layer

Histopathologic analysis of the two corneal buttons from the proband revealed an irregular, layered structure of corneal epithelium in which eosinophilic deposits were noted predominantly beneath the epithelium and involved the anterior stroma within a small range by hematoxylin and eosin staining (Fig. 3a). The presence of subepithelial congo-red-positive deposits that did not stained with Periodic acid-Schiff and Masson trichrome was also illustrated (Fig. 3b, c, d). UBM and AS-OCT scan of the proband demonstrated markedly increased reflectivity, due to deposits within the lesions and irregular epithelial thickening induced by corneal opacities which project into anterior stroma (Fig. 3e, f).

### Genotyping

**Fig. 4:**
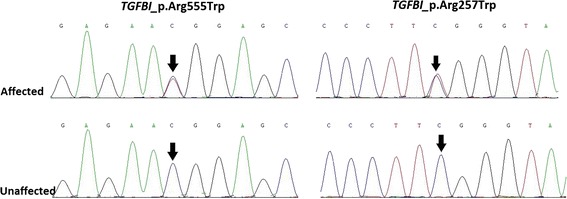
DNA sequence chromatograms of the proband. Note the heterozygous C > T nucleotide change in exon 12 and 6 of *TGFBI* gene (see the arrow) which altered the Arg to Trp and an unaffected individual (II:5) shows Arg at the same codon 555 and 257, respectively

Directional DNA sequencing revealed that affected members in this pedigree harbored a heterozygous missense mutation (Fig. 4), consisting of a C to T transition at nucleotide position 1663 at the first position of codon 555 in *TGFBI* exon 12 (c. 1663C > T), resulting in the substitution of a highly conserved Arginine by Tryptophan in the encoded TGFBIp (p. Arg555Trp). With the cosegregation of unaffected individuals and absence of this nucleotide change in 150 normal controls, we strongly assume that this mutation is pathological and exclude the possibility of its being a polymorphic change. The affected members were also heterozygous for three previously described, inconsequential, silent, single-nucleotide polymorphisms (SNPs) in exon 8, 11, 12, which did not affect the protein encoded (c.1142G > A(P.(=)), c.1577 T > C(P.(=)), c.1781C > T(P.(=)). Interestingly, in this pedigree, all patients were heterozygous for another rare variant in exon 6 of the *TGFBI* gene (p. Arg257Trp), (Fig. 4), which also cosegregated well within this family. We screened 150 controls of exon 6 and found 1 normal control harbored the p. Arg257Trp alteration and, thus, we considered it to be a rare SNP that was not previously reported.

## Discussion

Thiel-Behnke corneal dystrophy is an autosomal dominant inheritable disorder in which bilateral characteristic gray-to-white “honeycomb” corneal opacities and recurrent corneal erosions appear. It was described as corneal dystrophy affecting the surface epithelium, Bowman layer/anterior basement membrane complex and superficial stroma of the cornea by Küchle and associates [9]. Visual impairment may initially be mild, and visual acuity progressively worsens as the opacities coalesce into dense plaques over time [9, 10]. Previous studies have repeatedly shown that the missense mutation (p.Arg555Gln) of the *TGFBI* gene was strongly associated with TBCD [3, 7, 10–13]. In the present study, the associated of p. Arg555Trp mutation with TBCD was an unexpected and interesting finding, because, to date, the p. Arg555Trp mutation was frequently known to be responsible for subjects in corneal granular dystrophy type I (GCD I) [4, 7, 8, 11]. Therefore, our result challenges the current knowledge of “phenotypic specificity” and has permitted broadening the phenotype-moleculation correlation range within *TGFBI*-linked CDs.

To our knowledge, TBCD is frequently confused with Reis-Bucklers corneal dystrophy (RBCD, OMIM 608470) [14, 15], which also affects Bowman’s layer and can be difficult to differentiate both clinically and histopathologically [9, 10]. Phenotypically, this pedigree had some features that overlap with both TBCD and RBCD. Features typically associated with TBCD include honeycomb-pattern corneal opacities, relatively moderate deterioration of vision [16], and slow postoperative rate of recurrence [17], as is shown in the findings of this present study. The pathology report of subepithelial eosinophilic, congo-red-positive, PAS and Masson trichrome-negtive deposits also supported the diagnosis of TBCD [5, 18]. Meanwhile, the early disease onset occurring in the first decade of life, in keeping with RBCD. Microscopically, these two diseases can be distinguishable through transmission electron microscopy (TEM) in which RBCD is characterized by extracellular crystalloid, rod-shaped bodies [14, 15] and on the other hand, TBCD exhibits ultrastructurally “curly” fibers that stain positive for keratoepithelin [19]. Unfortunately we did not conduct TEM analysis as the tissue samples were not available, which was the limitation of our study.

What was interesting in our study is that a second heterozygous variant (p. Arg257Trp) also cosegregated with this family. Although has been confirmed to be a rare SNP, it was also predicted to change Arginine to Tryptophan in the Fas2 domain of encoded TGFBIp (NCBI Conserved Domain Database). To date, no pathogenic alterations have been found in the Fas2 region and there is no prior evidence that p. Arg257Trp alteration has any functional role, therefore, it is possible that sequence variations, especially the replacement of one residue with another, are tolerated and perhaps even modify the expression of the p. Arg555Trp mutation in this family. An effect of a yet-to-be-identified modifier genes remains a possibility. Further investigation is warranted of the mechanism of the underlying pathogenesis and an analysis of a larger cohort of families is necessary to determine whether the p. Arg257Trp polymorphism is in a linkage disequilibrium with the p. Arg555Trp mutation.

Severe visual deterioration induced by insoluble deposits of TBCD may be curable by superficial keratectomy, lamellar keratoplasty, penetrating keratoplasty (PKP), and phototherapeutic keratectomy (PTK) [20]. In our report, lamellar keratoplasty was successfully performed on the proband with a promising postoperative result.

## Conclusion

In conclusion, our study identified a novel phenotype-genotype correlation between p. Arg555Trp and TBCD. The genetic, clinical, and pathological finding described here broaden the spectrum of *TGFBI*-linked CDs. Analysis of additional corneal dystrophy cases in different population groups will facilitated the thorough understanding of this disease.

## Abbreviations

Arg: Arginine
Trp: Tryptophan
Gln: Glutamine
His: Histidine
Cys: Cysteine
Leu: Leucine
TGFBI: Transforming growth factor-beta induced
TBCD: Thiel-Behnke corneal dystrophy
RBCD: Reis-Bücklers corneal dystrophy
SNP: Single nucleotide polymorphism
CDs: Corneal dystrophies
GCD1: Granular corneal dystrophy type I
GCD2: Granular corneal dystrophy type II
LCD1: Lattice corneal dystrophy type 1
UBM: Ultrasound biomicroscopy
AS-OCT: Anterior-segment optical coherence tomography
H&E: Hematoxylin and eosin
PAS: Periodic acid-Schiff
PCR: Polymerase chain reaction
BCVA: Best Corrected Visual Acuity
UCVA: Uncorrected Visual Acuity
TEM: Transmission electron microscopy
PTK: Phototherapeutic keratectomy
